# Supplementary material for: Causal Relationship between Aging and Anorexia Nervosa: A White-Matter-Microstructure-Mediated Mendelian Randomization Analysis
Source: Biomedicines. 2024 Aug 16;12(8):1874. doi: 10.3390/biomedicines12081874 (PMC11351342; doi:10.3390/biomedicines12081874)
Supplement: Supplementary file 1 [file biomedicines-12-01874-s001.zip › Supplementary Figure/Figure S8/Leaveoneout plot of MR analysis for TL to ubm-b-1637.pdf]

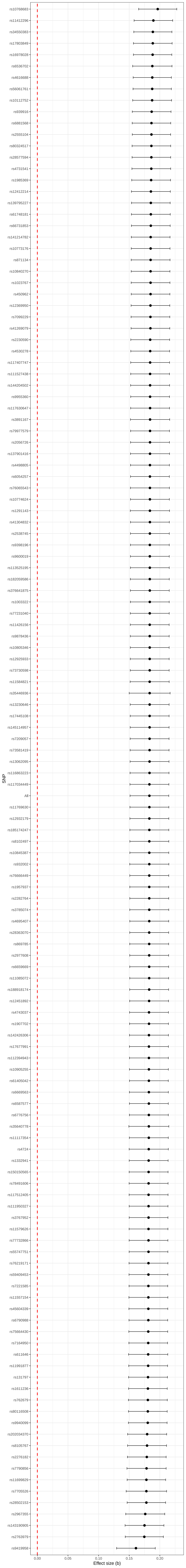

SNP
rs10768683
rs11412296
rs34550383
rs17803849
rs16978028
rs6536702
rs4616688
rs56061761
rs10112752
rs939916
rs6881568
rs2555104
rs80324517
rs28577594
rs4731541
rs1985369
rs12412214
rs139795227
rs61748181
rs66731853
rs141214782
rs10773176
rs871134
rs10840270
rs1023767
rs450962
rs12369950
rs7099229
rs41269079
rs2230590
rs4530278
rs117407747
rs111527438
rs144204502
rs9955360
rs117630647
rs3891167
rs79977579
rs2056726
rs137901416
rs4498805
rs6054257
rs76065543
rs10774624
rs1291143
rs41304832
rs2538745
rs9398196
rs9600019
rs113525195
rs182059586
rs376641875
rs1003322
rs77231040
rs11426156
rs9878436
rs10805346
rs12925933
rs73730598
rs11584821
rs35446936
rs13230646
rs17445108
rs145114957
rs7209057
rs73581419
rs13062095
rs116863223
rs117034449
All
rs11769630
rs12932179
rs185174247
rs8102497
rs10845387
rs932002
rs76666449
rs1957937
rs2282764
rs3785074
rs4695407
rs28363070
rs869785
rs2977608
rs6659669
rs11085072
rs188918174
rs12451892
rs4743037
rs1907702
rs142426306
rs17677991
rs112394943
rs10905255
rs61405042
rs6669563
rs6587577
rs6776756
rs35640778
rs11117354
rs4724
rs1332941
rs150150565
rs78491606
rs117512405
rs111950327
rs3767952
rs11579626
rs77732866
rs55747751
rs76219171
rs59409453
rs7221585
rs11557154
rs45604339
rs6790988
rs75664430
rs7164950
rs611646
rs11991877
rs131797
rs1611236
rs762679
rs80116508
rs9940099
rs202034370
rs8105767
rs2276182
rs7790856
rs11699829
rs7705526
rs28502153
rs2967355
rs143190905
rs2763979
rs9419958
0.00
0.05
0.10
0.15
0.20
Effect size (b)
